# Identification of Niche-Specific Gene Signatures between Malignant Tumor Microenvironments by Integrating Single Cell and Spatial Transcriptomics Data

**DOI:** 10.3390/genes14112033

**Published:** 2023-10-31

**Authors:** Jahanzeb Saqib, Beomsu Park, Yunjung Jin, Junseo Seo, Jaewoo Mo, Junil Kim

**Affiliations:** School of Systems Biomedical Science, Soongsil University, 369 Sangdo-Ro, Dongjak-Gu, Seoul 06978, Republic of Korea; jahanzeb@soongsil.ac.kr (J.S.); qpzm1367@soongsil.ac.kr (Y.J.); flashboxer@naver.com (J.M.)

**Keywords:** single-cell RNA sequencing, spatial transcriptomics, tumor microenvironments, data integration, niche-specific genes, spatial correlation

## Abstract

The tumor microenvironment significantly affects the transcriptomic states of tumor cells. Single-cell RNA sequencing (scRNA-seq) helps elucidate the transcriptomes of individual cancer cells and their neighboring cells. However, cell dissociation results in the loss of information on neighboring cells. To address this challenge and comprehensively assess the gene activity in tissue samples, it is imperative to integrate scRNA-seq with spatial transcriptomics. In our previous study on physically interacting cell sequencing (PIC-seq), we demonstrated that gene expression in single cells is affected by neighboring cell information. In the present study, we proposed a strategy to identify niche-specific gene signatures by harmonizing scRNA-seq and spatial transcriptomic data. This approach was applied to the paired or matched scRNA-seq and Visium platform data of five cancer types: breast cancer, gastrointestinal stromal tumor, liver hepatocellular carcinoma, uterine corpus endometrial carcinoma, and ovarian cancer. We observed distinct gene signatures specific to cellular niches and their neighboring counterparts. Intriguingly, these niche-specific genes display considerable dissimilarity to cell type markers and exhibit unique functional attributes independent of the cancer types. Collectively, these results demonstrate the potential of this integrative approach for identifying novel marker genes and their spatial relationships.

## 1. Introduction

The hallmark of cancer is not only characterized by isolated cancer cells but also by their interactions with neighboring normal cells, including immune cells, fibroblasts, and blood vessels; collectively, they form the tumor microenvironment (TME). These interactions play vital roles in promoting tumor growth, angiogenesis, invasion of nearby tissues, and evasion of immune surveillance [1]. For instance, cancer cells employ negative regulatory molecules such as CTLA4 and PD-1 to suppress immune responses or evade detection [2]. Moreover, cell–cell communications between cancer cells and surrounding stromal cells actively promote tumor growth and metastasis [3]. Understanding these intricate cell–cell interactions within the TME is practically important [4,5,6,7], because it facilitates the development of targeted therapies aimed at disrupting these communication networks, ultimately enhancing cancer treatment outcomes.

Technological advances in next-generation sequencing have facilitated preventive oncological practices at molecular resolution [8]. Furthermore, the unbiased and systematic characterization of the cellular transcriptomes in each tissue can be achieved by employing single-cell RNA sequencing (scRNA-seq) [9]. Using scRNA-seq for tumor samples, many studies have discovered various cellular subpopulations and emphasized intercellular communications in various cancer types such as glioblastoma [10,11], oligodendroglioma [12], melanoma [13], glioma [14,15], breast cancer [16], prostate cancer [17], head and neck cancer [18], hepatocellular carcinoma [19,20,21], colorectal cancer [22], and lung adenocarcinoma [23].

Despite the power of scRNA-seq in oncology, spatial information is lost because of tissue dissociation before sequencing, thereby restricting our understanding of cellular interactions and neighboring structures in the TME. To resolve the spatial composition of tissues based on cell type, various sequencing-based spatial transcriptomics technologies have been developed, including ST [24], 10X Visium, high-definition spatial transcriptomics (HDST) [25], Slide-seq [26,27], Seq-scope [28], and DBiT-seq [29]. Using ST, Berglund et al. investigated the spatial maps of prostate cancer transcriptomes to distinguish between healthy and diseased areas and observed gene expression changes during prostate cancer growth [30]. Furthermore, using ST technology, Thrane et al. revealed the detailed landscape of melanoma metastases [31].

However, spatial transcriptomics has limitations because it does not provide cellular resolution [32]. The 10X Visium platform, a widely used spatial transcriptomics technique, can capture the transcriptomes of 10–50 cells depending on cell size. Recently developed high-resolution technologies such as HDST, Slide-seq, Seq-scope, and DBiT-seq have improved resolution at the subcellular level. Nevertheless, these technologies can still capture multiple cells due to the three-dimensional organization of tissues and are constrained by cost and usability [33,34].

To address these limitations, bioinformaticians have suggested and developed several algorithms for integrating scRNA-seq and spatial transcriptomics data to enhance spatial resolution [35]. Tangram [36] uses a deep learning framework and non-convex optimization for the spatial alignment of scRNA-seq data, thereby enabling the mapping of cells from the scRNA-seq data to spots of the spatial transcriptomic data. To determine cell composition, robust cell type decomposition (RCTD) algorithm [37] uses supervised learning based on a probabilistic model; Cell2location [38] estimates the abundance of each cell type using a Bayesian model based on negative binomial regression; SpatialDWLS [39] adopts the weighted least squares method; Stereoscope [40] takes advantage of the model-based probabilistic technique; and SPOTlight [41] employs seeded non-negative matrix factorization.

In this paper, we introduced NicheSVM, a user-friendly framework to perform single-cell and spatial transcriptomics. NicheSVM uses the support vector machines (SVMs) algorithm for deconvolution, followed by niche-specific gene analysis. Originally developed for physically interacting cell sequencing (PIC-seq) analysis with matched scRNA-seq [42], we adapted the same algorithms for 10X Visium data and created a graphical user interface (GUI) using MATLAB. First, NicheSVM integrates scRNA-seq and spatial transcriptomic data by exploiting multi-class SVMs. Statistical analysis identified the distinct gene sets, particularly the compositions of their neighbors within a spot. In our previous study of PIC-seq analysis, we identified neighbor-specific genes that were enriched when the two cell types were in contact with each other. Herein, we introduced the concept of “niche-specific genes”, whose expression was enhanced when two different cell types were colocalized within each spot of the Visium platform. We applied NicheSVM to the datasets of five cancer types including breast cancer (BRCA), gastrointestinal stromal tumor (GIST), liver hepatocellular carcinoma (LIHC), ovarian cancer (OVCA), and uterine corpus endometrial carcinoma (UCEC) [43]. Our comprehensive analysis using the NicheSVM pipeline revealed unique niche-specific gene sets that were distinct from the cell type-specific genes. Furthermore, we discovered that the niche-specific genes exhibited higher cross-correlation values for consecutive neighboring spots than cell type-specific genes, indicating that niche-specific genes are more involved in cell–cell interactions. In conclusion, our method provides new insights into cell–cell communications in complex tissues.

## 2. Materials and Methods

### 2.1. Preparation of Public Dataset of Matched or Paired scRNA-Seq and Visium Data

A public dataset of matched or paired scRNA-seq and Visium data was downloaded for five different cancer types (BRCA, GIST, LIHC, OVCA, and UECE) [43]. Supplementary Table S1 lists the pairing information of Visium slides and scRNA-seq data. For the scRNA-seq data, we adopted Harmony [44] for the integration of the various samples of all five cancer types based on the preprocessed data provided in the original paper. In the original paper, the authors analyzed the scRNA-seq data using conventional Seurat pipeline [45].

### 2.2. Implementation of NicheSVM-GUI

The NicheSVM, which was originally developed for PIC-seq, was used to accommodate 10X Visium data. To make it more user-friendly, a GUI was developed using MATLAB R2023a. This NicheSVM-GUI is freely available and compatible with MATLAB Runtime. Utilizing this GUI, users can simplify the analysis process, facilitating the integration and visualization of cell type markers and niche-specific genes.

### 2.3. Enrichment Analysis

Enrichr [46] was used to identify the enriched Gene Ontology (GO) terms and Kyoto Encyclopedia of Genes and Genomes (KEGG) pathways associated with the niche-specific genes and cell type markers.

### 2.4. Spatial Cross-Correlation Analysis

Spatial cross-correlation was used to elucidate the relationship between gene pairs within a TME. We calculated the two-dimensional (2D) cross-correlation of two genes’ log normalized expression, ***A*** and ***B***, in a Visium slide of size *M*-by-*N* as follows:

(1) $$C\left(k,l\right)=\sum_{m=0}^{M-1}\sum_{n=0}^{N-1}A\left(m,n\right)\;B(m-k,n-l).$$

where *k* and *l* represent the number of shifts in the x- and y-coordinates, respectively. The 2D cross-correlation for a distance of 1 was specifically defined as the average of six cross-correlation values obtained by shifting in six different directions at a distance of 1 because the spots are arranged in a hexagonal grid.

### 2.5. Survival Analysis Based on the Cancer Genome Atlas (TCGA) Data

Survival analysis was conducted using bulk RNAseq data of five cancer types from TCGA. The Kaplan–Meier method implemented in R was used.

### 2.6. Statistical Tests

Two-sided Student’s *t*-tests were performed to compare the spatial cross-correlation values and the *p*-values obtained from the survival analysis of the niche-specific genes with those of the cell type markers.

## 3. Results

### 3.1. NicheSVM Algorithm

**Figure 1:**
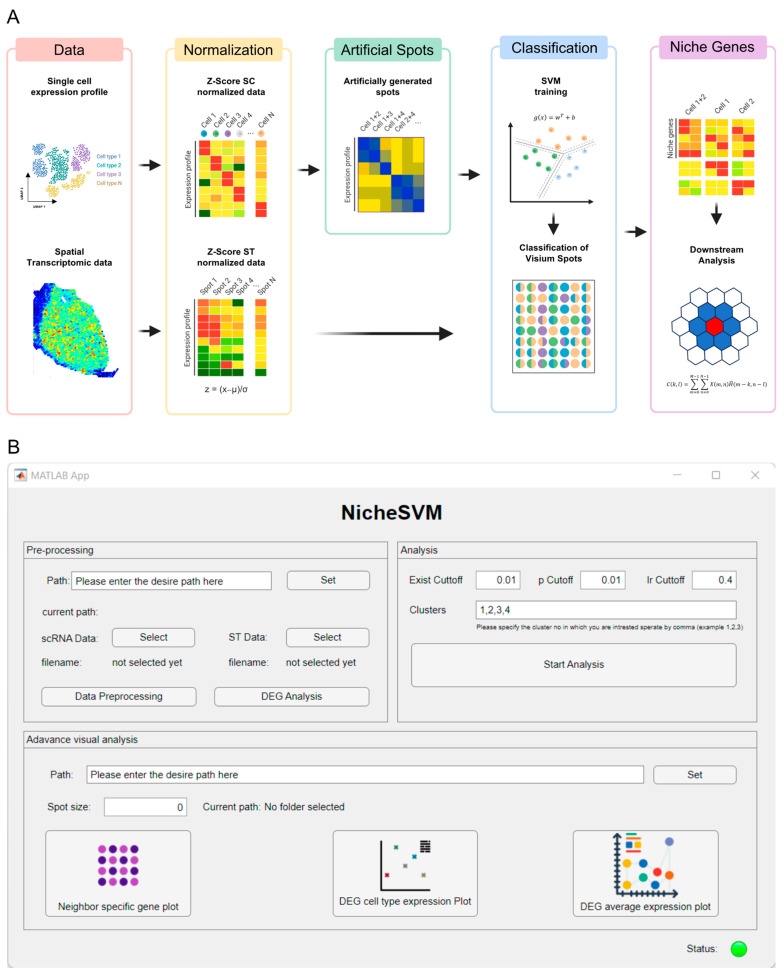
**NicheSVM algorithm.** (**A**) scRNA-seq data with cell type information and spatial transcriptomics data from the same tissue sample are the input for NicheSVM. The algorithm comprises the following steps: (1) z-score normalization is applied to both datasets; (2) artificial spots are generated for every combination of two cell types by averaging the randomly selected z-score of single cell expression; (3) multi-class SVMs are trained using the artificial spatial data; (4) the spatial transcriptomics data are classified into the respective cell type combinations; and (5) niche-specific genes are identified by comparing classified spatial data and matched artificial spatial data. (**B**) Graphical user interface for NicheSVM.

Figure 1A illustrates the analysis pipeline of NicheSVM, which comprises of five distinct phases for gaining insights into the underlying biological processes in a tissue sample. Both the analyzed scRNA-seq data with cell type information and spatial transcriptomic data from the same types of samples are required as input data for NicheSVM. scRNA-seq data provide information on gene expression at the individual cell level; on the other hand, spatial transcriptomic data offer spatial context by capturing gene expression patterns in the tissue. To integrate these two modalities, we assumed that z-value normalization could ignore platform dependency. This assumption is confirmed through principal component analysis (PCA) conducted on raw counts, log normalized counts, and z-scores of the BRCA dataset (Supplementary Figure S1). The scatter plot of PCA demonstrates that scRNA-seq and Visium data overlapped when the PCA was performed solely on z-scores. Based on this, we transformed each dataset independently.

Second, 10,000 artificial spots are generated for every combination of two cell types by averaging the z-scores of single cell expression of two randomly selected cells. For simplicity, we assumed that only two major cell types could span the expression patterns of each spot of Visium data. Third, multi-class SVMs were trained using the expression data of the artificial spots. Multi-class SVMs include multiple SVM binary learners in a one versus one design. To avoid cell type biases, the top five differentially expressed genes (DEGs) for each cell type were used. Fourth, the spatial transcriptomics data were classified into the respective cell type combinations. Finally, niche-specific genes that represent the genes expressed in some microenvironments or cellular niches were identified. Niche-specific genes were identified by comparing the z-score of spatial data with artificial spatial data generated by averaging randomly selected z-score of single cell expression for each combination using two-sided Wilcoxon’s rank-sum test. A GUI was generated in the pipeline (Figure 1B).

### 3.2. NicheSVM Identified Niche-Specific Genes in Five Cancer Types

To investigate the expression of the niche-specific genes between different cell types within local areas of cancer tissues, we used the publicly available paired or matched scRNA-seq and Visium data of five cancer types (BRCA, GIST, LIHC, OVCA, and UCEC) [43]. To construct references for cell type expression, the cell type annotations provided in the original study were used. Across these five cancer types, we found 11 common cell types, including B cells (BC), dendritic cells (DC), endothelial cells (EN), normal epithelial cells (NE), fibroblasts (FB), macrophages (MAC), malignant cells (MAL), neutrophils (NE), natural killer cells (NK), T cells (TC), and tissue stem cells (TSC) (Supplementary Table S2). Chondrocytes (CH) were detected in four cancer types, except for LIHC, whereas smooth muscle cells (SMC) were detected in all four cancer types, except for UCEC.

**Figure 2:**
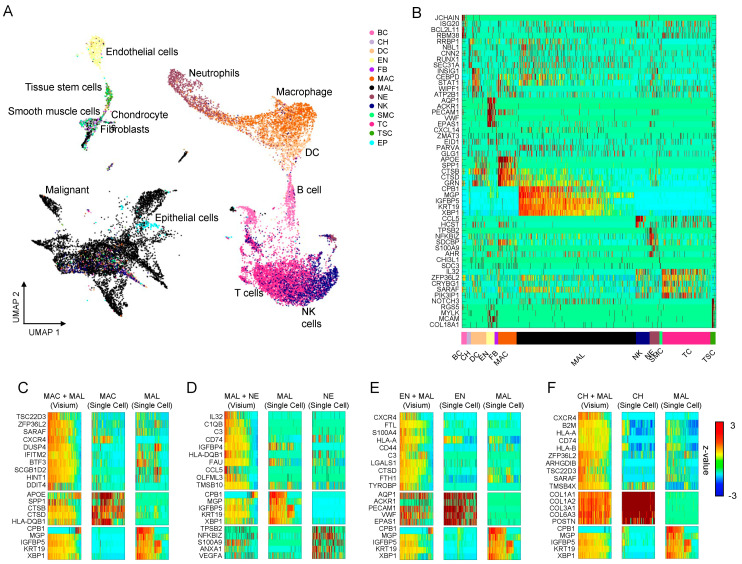
**NicheSVM reveals niche-specific genes in breast cancer** (**BRCA**)**.** (**A**) Uniform manifold approximation and projection representation showing heterogeneous cell types in five cancer types: BRCA, gastrointestinal stromal tumor, liver hepatocellular carcinoma, ovarian cancer, and uterine corpus endometrial carcinoma. (**B**) Heatmap visualizing the cell type markers for each cell type in scRNA-seq data of BRCA. (**C**–**F**) Heatmaps depicting the top 10 niche-specific genes and their corresponding cell type markers in the Visium and scRNA-seq data for four major combinations: MAC + MAL (**C**), MAL + NE (**D**), EN + MAL (**E**), and CH + MAL (**F**) of BRCA.

Figure 2A illustrates the Uniform Manifold Approximation and Projection (UMAP) representation of cell types derived from the scRNA-seq data of five cancer types (Figure 2A and Supplementary Figure S2). This visualization clearly demonstrates well-separated cell clusters. To confirm the distinctiveness of each cell type, we identified cell type marker genes using the Wilcoxon’s rank sum test (Supplementary Tables S3–S7). These identified markers exhibited unique expression profiles (Figure 2B), indicating the presence of distinguishable transcriptomic features in each cell type. For example, MGP, a known marker for BRCA malignancy, is highly expressed in BRCA cells [47].

Using these well-defined cell type expression profiles, we constructed the NicheSVM pipeline. The cell types having less than 10 cell type markers were excluded for this pipeline. This process allowed us to predict cell type combinations within each Visium spot across all five cancer types (Supplementary Figure S3). The error rates of 10-fold cross-validation for the SVMs were 12.34%, 19.61%, 8.74%, 15.15%, and 12.86% for BRCA, GIST, LIHC, OVCA, UCEC, respectively, which are lower than the error rate for PIC-seq data, which was between 17–29%. This suggests that the NicheSVM algorithm is suitable for analyzing Visium data.

Subsequently, we identified niche-specific genes by comparing the niche combinations of the Visium spots with artificial Visium spots generated from scRNA-seq data (Figure 2C–F). We focused on the niche combinations mediated by malignant cells because they provide insights into the relationship between cancer cells and their TMEs. Figure 2C–F display the top 10 niche-specific genes for four niche combinations: MAL + MAC, MAL + NE, MAL + EN, and MAL + CH. Interestingly, some genes associated with the major histocompatibility complex (MHC), such as CD74, B2M, HLA-A, and HLA-B, were highly expressed in the CH + MAL niche combination spots (Figure 2F). Using the same pipeline, we identified niche-specific gene sets in the other four cancer types (Supplementary Figures S4–S7).

### 3.3. Niche-Specific Genes in Five Cancer Types Exhibit Unique Characteristics Different from Cell Type Markers

**Figure 3:**
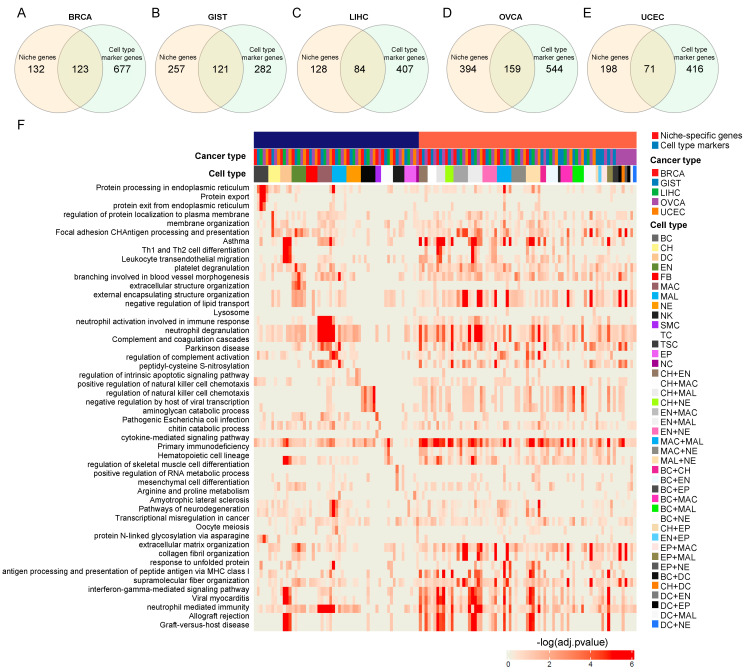
**Niche-specific genes in five cancer types exhibit unique characteristics different from cell type markers.** (**A**–**E**) Venn diagram showing the comparison between niche-specific and cell type markers in five cancer types: BRCA, breast cancer (**A**); GIST, gastrointestinal stromal tumor (**B**); LIHC, liver hepatocellular carcinoma (**C**); OVCA, ovarian cancer (**D**); and UCEC, uterine corpus endometrial carcinoma (**E**). (**F**) Comprehensive gene set enrichment analysis revealing the distinctive functional attributes of niche-specific genes from cell type markers in five cancer types.

In the previous section, we identified the niche-specific genes in five cancer types. We hypothesized that niche-specific genes encapsulate unique aspects of cancer hallmarks that are distinct from cell type markers. To validate this hypothesis, we selected the top 50 niche-specific genes identified in each combination (Supplementary Tables S8–S12) and compared them with cell type markers. The Venn diagrams in Figure 3A–E summarize that approximately 50–75% of the niche-specific genes do not overlap with cell type markers across five cancer types, representing the uniqueness of niche-specific genes. Furthermore, we compared the niche-specific genes with cell type markers in a group-by-group manner using hypergeometric tests; we observed no significant association between these two categories (Supplementary Figure S8).

Subsequently, we analyzed the enriched KEGG and GO terms for both cell type markers and niche-specific genes (Figure 3F). This comprehensive comparison highlighted the differences between these two categories. Interestingly, the enriched terms for cell type markers varied based on different cell types, whereas the enriched terms for niche-specific genes shared many commonalities, including “cytokine-mediated signaling pathway” and “antigen processing and presentation” across all five cancer types. Collectively, these results suggest the presence of common characteristics in niche-specific genes in different cancer types, emphasizing their association with cell–cell interactions.

### 3.4. Niche-Specific Genes Are More Spatially Correlated with Each Other

**Figure 4:**
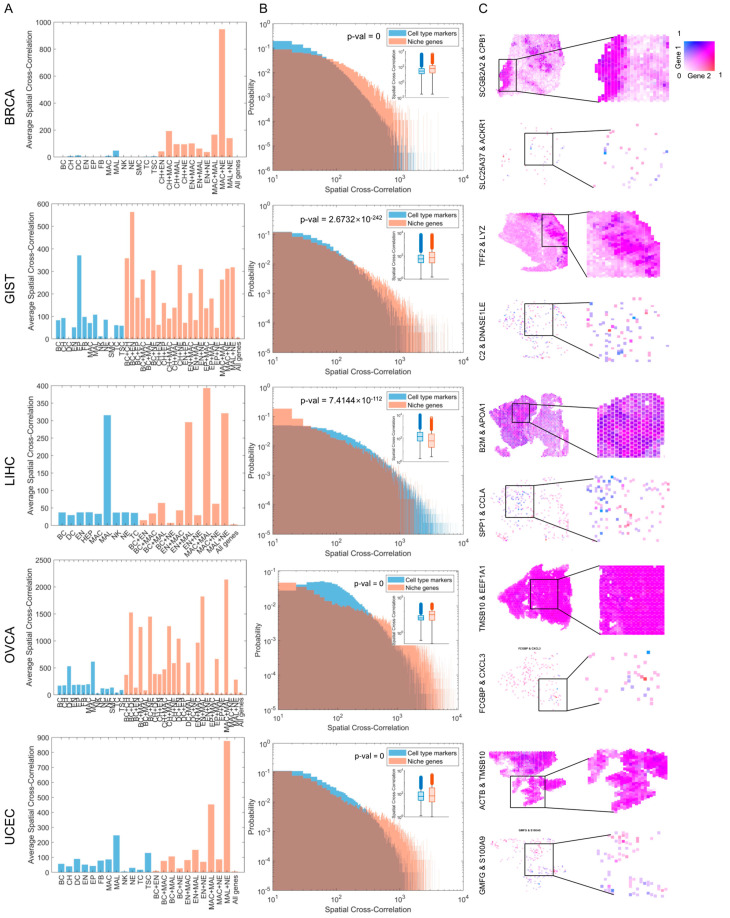
Niche-specific genes are more spatially cross-correlated to each other compared with cell type markers in five cancer types. (**A**) Bar plots showing the average spatial cross-correlation values for each gene group of niche-specific genes and cell type markers. (**B**) Distributions of the spatial cross-correlation of the niche-specific genes and cell type markers. The spatial cross-correlation values of niche-specific genes are significantly higher than those of cell type markers in five cancer types. (**C**) Examples of gene pairs with high spatial cross-correlation values and negative controls (low cross-correlation).

In our functional analysis, we observed that the niche-specific genes may be significantly associated with cell–cell interactions. This finding led us to hypothesize that these niche-specific genes may also have a spatial correlation, meaning that they can be detected close to each other within the tissue. To test this hypothesis, we determined the spatial cross-correlations (Figure 4) between all pairs of cell type markers within each cell type and between all pairs of niche-specific genes within each niche combination. We considered the nearest neighbors when determining spatial correlations. Surprisingly, across all five cancer types, pairs of niche-specific genes exhibited higher spatial correlations than those of cell type markers (Figure 4A). To further validate this trend, we conducted the Student’s *t*-tests between the spatial correlation values of all pairs of niche-specific genes and those of cell type markers. We observed a significantly higher spatial correlation in niche-specific genes for BRCA, GIST, OVCA, and UCEC (Figure 4B). However, for LIHC, the spatial correlation was higher for cell type markers than for niche-specific genes. Notably, the elevated spatial correlations of cell type markers in LIHC primarily stem from the markers associated with malignant cells. In general, many niche-specific genes in LIHC displayed high spatial correlations.

Figure 4C presents some examples of niche-specific gene pairs that exhibited high spatial correlations. For instance, in a BRCA tissue sample, SCGB2A2 and CPB1 were highly spatially correlated. Although the relationship between these two genes remains unknown, both are associated with BRCA. In a previous study, immunohistochemistry (IHC) staining revealed SCGB2A2 as a marker for bone marrow micrometastases in BRCA [48]; on the other hand, proteomics and IHC staining revealed CPB1 as a potential metastasis marker for BRCA [49]. In GIST, we observed that TFF2 and LYZ had the highest correlations. Interestingly, these two genes are simultaneously upregulated in TP53/ARID1A double-knockout human organoids, which serve as a model for gastric cancer malignancy [50]. These findings suggest that our approach for identifying niche-specific genes can contribute to the discovery of new cancer markers. We also validated the niche-specific genes using a high-resolution spatial transcriptomics MERSCOPE obtained from a BRCA sample [51]. We calculated Pearson’s correlation coefficients (PCCs) between niche-specific genes and their corresponding two cell type markers. The PCC values were higher when comparing the average of the two cell type markers to a niche-specific gene than when comparing each cell type marker separately (Supplementary Table S13 and Supplementary Figure S9).

To investigate the role of niche-specific genes in cancer progression, we performed a survival analysis using TCGA data for all five cancer types (Supplementary Figure S10). We observed that the *p*-values associated with niche-specific genes were not significantly different from those associated with cell type markers. However, in the case of BRCA, the *p*-values for niche-specific genes were notably higher than those for cell type markers (Supplementary Figure S10A). This suggests that niche-specific genes considerably affect the survival of patients with BRCA. The examples presented in Supplementary Figure S9B for genes such as CD52 and HLA-DRA illustrate this point. This implies that niche-specific genes may have important clinical implications for BRCA. Furthermore, we investigated the correlation between the niche-specific gene expression and drug treatment efficiency using Cancer Cell Line Encyclopedia (CCLE) RNAseq and drug efficiency dataset [52]. We selected two anti-cancer drugs, BVD-523 and OXALIPLATIN, with the highest number of available RNAseq data, identifying highly correlated niche-specific genes with IC50 (half maximal inhibitory concentration) of the two anti-cancer drugs across five cancer types (Supplementary Figure S11). We also examined the relationships between niche-specific gene expression and cancer stages for four cancer types, and the results showed that CYBRD1, ENO1, CREB1, and ESR1 were significantly associated with the cancer stage of BRCA, LIHC, OVCA, and UCEC, respectively (Supplementary Figure S12).

## 4. Discussion

Our proposed algorithm, NicheSVM, uses an integrative approach to harmonize spatial transcriptomics data with scRNA-seq data to identify niche-specific genes. Five cancer types, namely, BRCA, GIST, LIHC, OVCA, and UCEC, were considered in the paired and matched scRNA-seq and ST datasets to investigate the validity of the proposed method. In particular, the proposed analysis pipeline successfully revealed distinct niche-specific gene signatures from cell type markers. Moreover, the obtained niche-specific gene signatures displayed enhanced spatial relationships between neighboring spots.

SVM-based PIC-seq data analysis has been successfully used to identify neighbor cell-dependent gene expression during mouse embryonic development, which was validated by in vitro coculture experiments [42]. This result indicates that SVM can help identify cell type combinations, providing neighboring cell-dependent genes. This success led us to adapt this analysis pipeline to 10X Visium, a spatial transcriptomics technology, to identify niche-specific genes affected by cell type combinations within 55 µm.

Comprehensive gene set enrichment analysis was conducted to determine the functional characteristics of niche-specific genes. Interesting similarities were observed across five cancer types for enriched terms, such as “antigen processing and presentation” and “cytokine-mediated signaling pathway”. This indicates that niche-specific genes play conserved roles in the regulation of immunological responses and cell–cell communications between nearby cells. Understanding these shared functions provides valuable insights regarding the pathways and prospective treatment targets essential for tumor development and progression. To confirm this functional relevance of the niche-specific genes in cell–cell communications, we conducted CellPhoneDB [53] analysis, a widely used scRNA-seq analysis tool based on a ligand–receptor database. The analysis revealed that the significant ligand–receptor pairs had minimal overlap with niche-specific genes (Supplementary Figure S13). This indicates that using NicheSVM on Visium data might have limitations due to the small number of features (top five cell type markers for SVM training), or it is possible that niche-specific genes cannot be identified solely using ligand–receptor information. To confirm whether the niche-specific genes depend on the number of features, we conducted NicheSVM with top 10 markers for BRCA dataset, identifying 265 niche-specific genes, which include 180 genes from the previous analysis (Supplementary Table S14).

In addition, we demonstrated that niche-specific genes exhibit higher spatial correlations than cell type markers. This suggests that these genes have similar expression patterns and are more localized and coordinated. This finding supports the idea that these genes are spatially coordinated within the tissue and play a vital role in cell–cell interactions. In sum, this demonstrates the significance of these genes in understanding tumor progression and microenvironmental dynamics.

Survival analysis using TCGA data revealed that niche-specific genes have a greater effect on patient survival than cell type markers in BRCA. This indicates that the expression of these niche-specific genes can act as potential prognostic indicators of BRCA. Further investigation of these genes and their clinical relevance may lead to the development of novel therapeutic targets for patients with BRCA.

The presented method is limited by the assumption that each Visium spot, constituting 10–50 cells, predominantly comprises only two major cell types. This assumption simplifies the overall communication mechanism among different cell types to reduce complexity. In real scenarios, Visium data may involve more than two cell types within a single spot. Expanding this approach to those multiple cell type combinations holds the potential to reveal novel niche-specific genes.

### 4.1. Code Availability

Source code and GUI file for NicheSVM are available at https://github.com/jahanzebsaqib/NicheSVM-GUI (accessed on 28 September 2023).

### 4.2. Key Points

- NicheSVM is a user-friendly analysis framework for identifying niche-specific genes based on scRNA-seq and Visium data.
- NicheSVM was applied to the paired and matched scRNA-seq and Visium data of five cancer types, revealing the niche-specific genes associated with cell–cell interactions.
- Niche-specific genes exhibit higher spatial correlation values than cell type-specific genes.

## 5. Conclusions

In this study, we introduce NicheSVM, a user–friendly framework for analyzing single cell and spatial transcriptomics. NicheSVM uses SVMs for deconvolution followed by niche-specific gene analysis. Our comprehensive analysis using the NicheSVM on matched scRNAseq and Visium platform across five cancer types revealed unique niche-specific gene sets associated with cell–cell interactions. In conclusion, our results suggest that integrative analysis using single cell and spatial transcriptomics enables to identify novel markers for the interactions between tumor cells and their microenvironments.

## Data Availability

Processed datasets are available at https://github.com/jahanzebsaqib/NicheSVM-GUI.

## Supplementary Materials

The following supporting information can be downloaded at: https://www.mdpi.com/article/10.3390/genes14112033/s1, Figure S1: Principal component analysis (PCA) demonstrated that z-score normalization can reduce platform difference between scRNAseq and Visium data obtained from breast cancer samples; Figure S2: Uniform manifold approximation and projection of the integrated scRNAseq data showing five cancer types and malignancy in different colors; Figure S3: Predicted cell type combinations within each Visium spot across all five cancer types; Figure S4: NicheSVM reveals niche-specific genes in GIST; Figure S5: NicheSVM reveals niche-specific genes in LIHC; Figure S6: NicheSVM reveals niche-specific genes in OVCA; Figure S7: NicheSVM reveals niche-specific genes in UCEC; Figure S8: The association between niche-specific genes and cell type markers across five cancer types; Figure S9: Validation of a niche-specific gene using high-resolution spatial transcriptomics data MERSCOPE obtained from a breast cancer sample; Figure S10: Survival analysis of top 50 niche-specific genes and cell type markers across five cancer types; Figure S11: Example of niche-specific genes showing high correlation with drug treatment efficiency in cancer cell line encyclopedia (CCLE) data; Figure S12: Example of niche-specific genes showing association with cancer stage in the cancer genome atlas (TCGA) data; Figure S13: Discrepancy between niche-specific genes and gene sets identified from CellPhoneDB a ligand-receptor analysis tool; Table S1: Single Cell RNA Seq and 10X Visium slides; Table S2: Number of cells in each sample; Table S3: Cell type markers of BRCA; Table S4: Cell type markers of GIST; Table S5: Cell type markers of LIHC; Table S6: Cell type markers of OVCA; Table S7: Cell type markers of UCEC; Table S8: Niche specific genes of BRCA; Table S9: Niche specific genes of GIST; Table S10: Niche specific genes of LIHC; Table S11: Niche specific genes of OVCA; Table S12: Niche specific genes of UCEC; Table S13: Niche-specific gene expression is validated in high-resolution spatial transcriptomics MERSCOP data; Table S14: Niche specific genes of BRCA (Training NicheSVM with top 10 DEGs for each cell type).
