# Supplementary material for: Identification of Niche-Specific Gene Signatures between Malignant Tumor Microenvironments by Integrating Single Cell and Spatial Transcriptomics Data
Source: Genes (Basel). 2023 Oct 31;14(11):2033. doi: 10.3390/genes14112033 (PMC10671538; doi:10.3390/genes14112033)
Supplement: Supplementary file 1 [file genes-14-02033-s001.zip › Supplementary_Figures.pdf]

Identification of niche-specific gene signatures between malignant and tumor microenvironments by integrating single cell and spatial transcriptomics data

Supplementary Figures

Jahanzeb Saqib, Beomsu Park, Yunjung Jin, Junseo Seo, Jaewoo Mo, Junil Kim*

School of Systems Biomedical Science, Soongsil University, 369 Sangdo-Ro, Dongjak-Gu, Seoul 06978, Republic of Korea

*To whom correspondence should be addressed to JK (Tel: +82-2-820-0452; E-mail: junilkim@ssu.ac.kr)

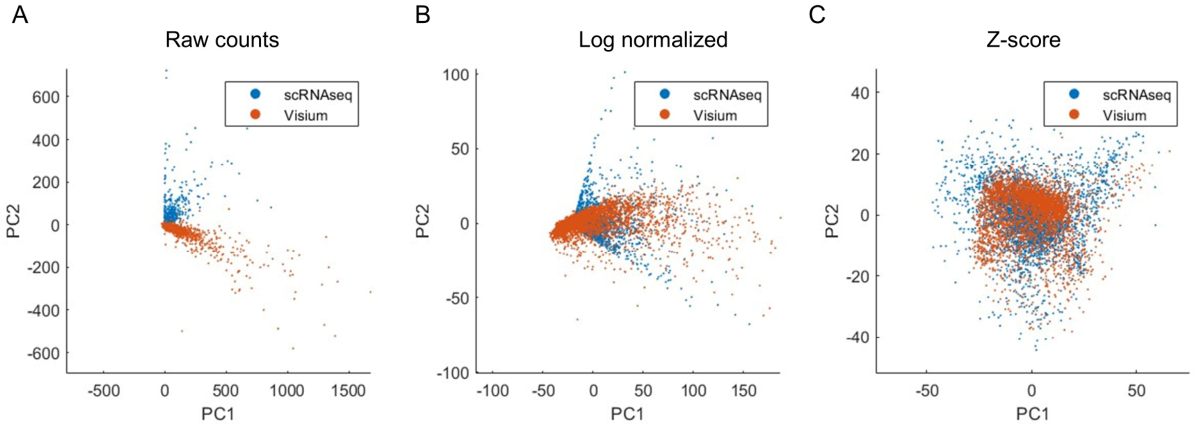

Supplementary Figure S1. Principal component analysis (PCA) demonstrated that z-score normalization can reduce platform difference between scRNAseq and Visium data obtained from breast cancer samples. PCA plots of (**A**) raw counts, (**B**) log normalized counts, and (**C**) z-scores of scRNAseq and Visium data.

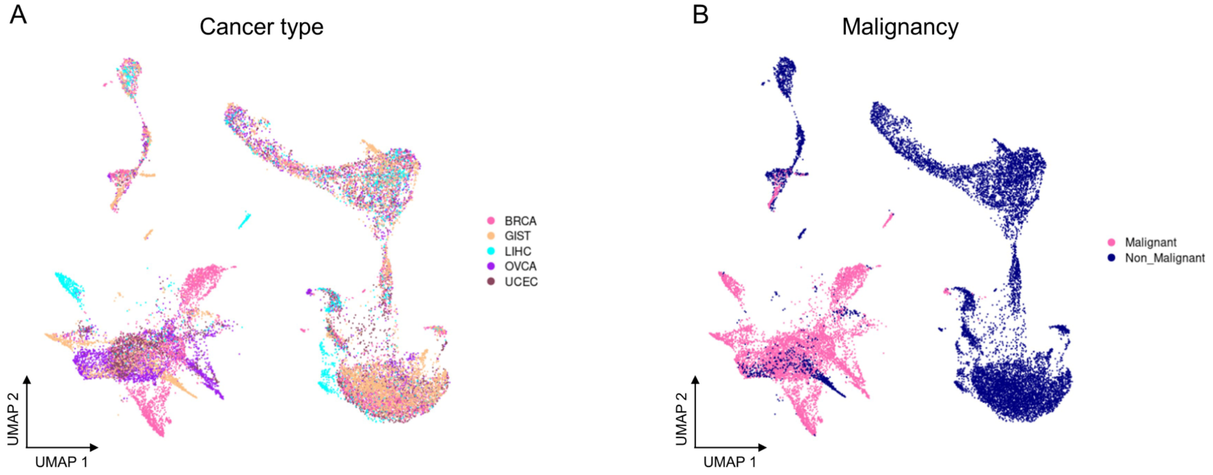

Supplementary Figure S2. Uniform manifold approximation and projection of the integrated scRNAseq data showing five cancer types (A) and malignancy (B) in different colors.

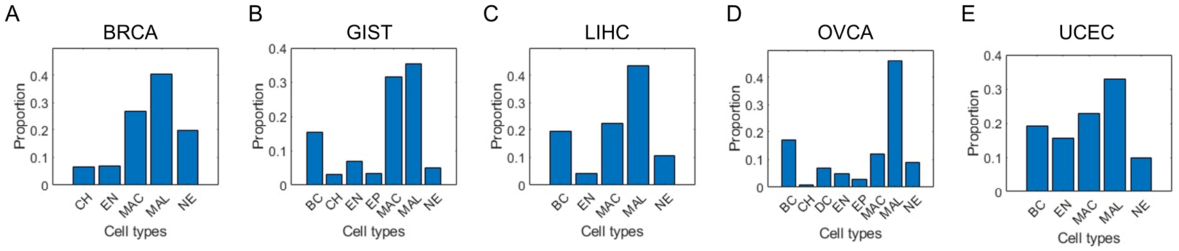

Supplementary Figure S3. Predicted cell type combinations within each Visium spot across all five cancer types. Proportion of cell types in Visium slides of BRCA; breast cancer (**A**), GIST; gastrointestinal stromal tumor (**B**), LIHC; liver hepatocellular carcinoma (**C**), OVCA; ovarian cancer (**D**), and UCEC; uterine corpus endometrial carcinoma (**E**).

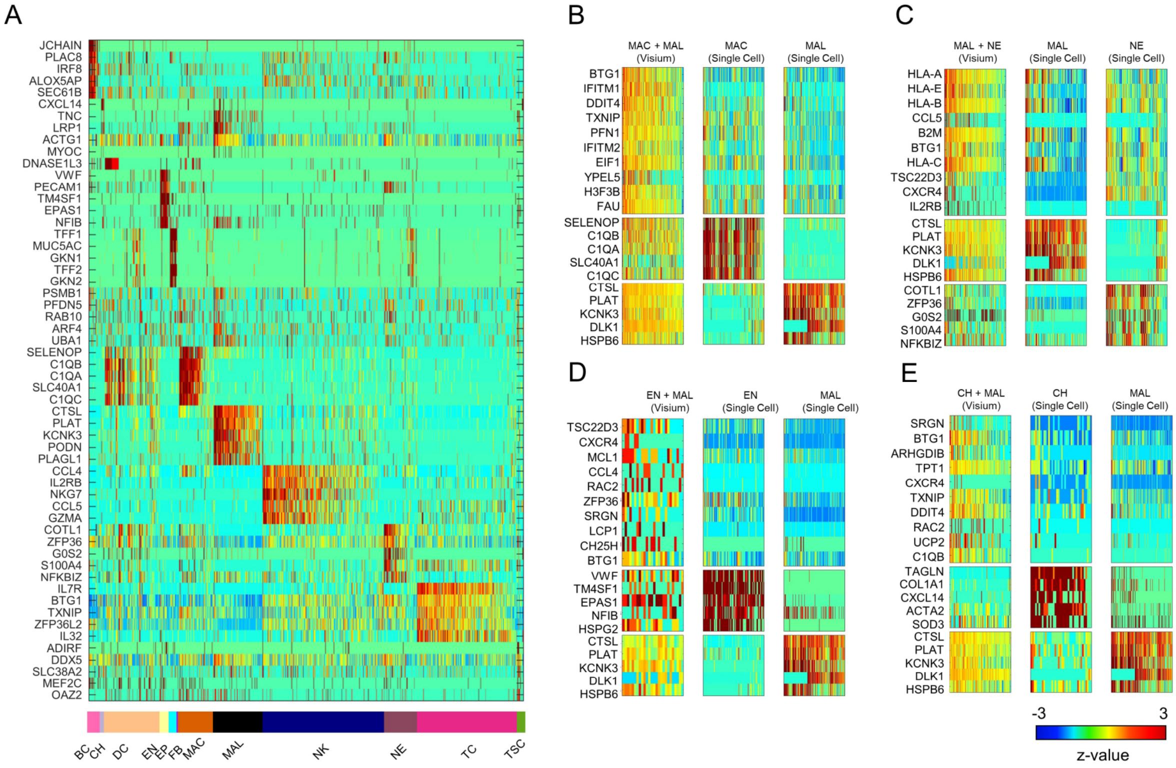

Supplementary Figure S4. NicheSVM reveals niche-specific genes in GIST. (**A**) Heatmap visualizing cell type markers for each cell type in scRNAseq data. (**B-E**) Heatmaps depicting the top 10 niche-specific genes and their corresponding cell type markers in Visium and scRNAseq data for four major combinations; MAC+MAL (**B**), MAL+NE (**C**), EN+MAL (**D**), and CH+MAL (**E**).

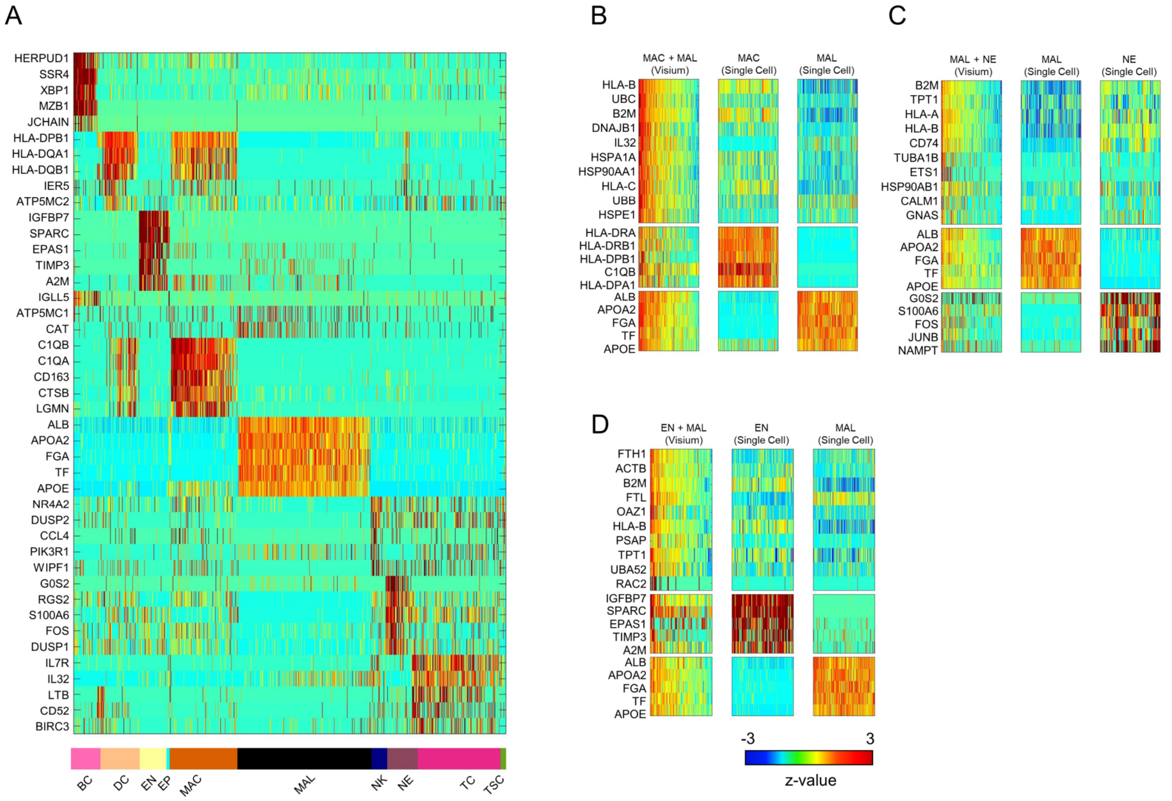

Supplementary Figure S5. NicheSVM reveals niche-specific genes in LIHC. (**A**) Heatmap visualizing cell type markers for each cell type in scRNAseq data. (**B-D**) Heatmaps depicting the top 10 niche-specific genes and their corresponding cell type markers in Visium and scRNAseq data for four major combinations; MAC+MAL (**B**), MAL+NE (**C**), and EN+MAL (**D**).

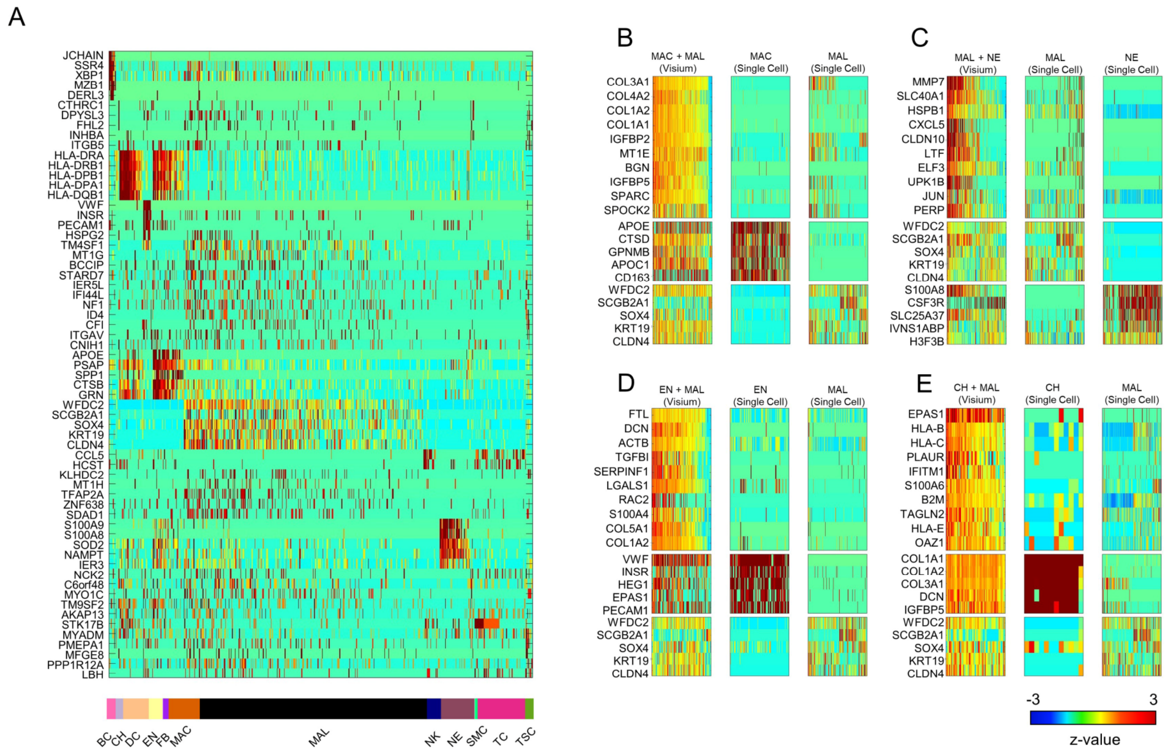

Supplementary Figure S6. NicheSVM reveals niche-specific genes in OVCA. (**A**) Heatmap visualizing cell type markers for each cell type in scRNAseq data. (**B-E**) Heatmaps depicting the top 10 niche-specific genes and their corresponding cell type markers in Visium and scRNAseq data for four major combinations; MAC+MAL (**B**), MAL+NE (**C**), EN+MAL (**D**), and CH+MAL (**E**).

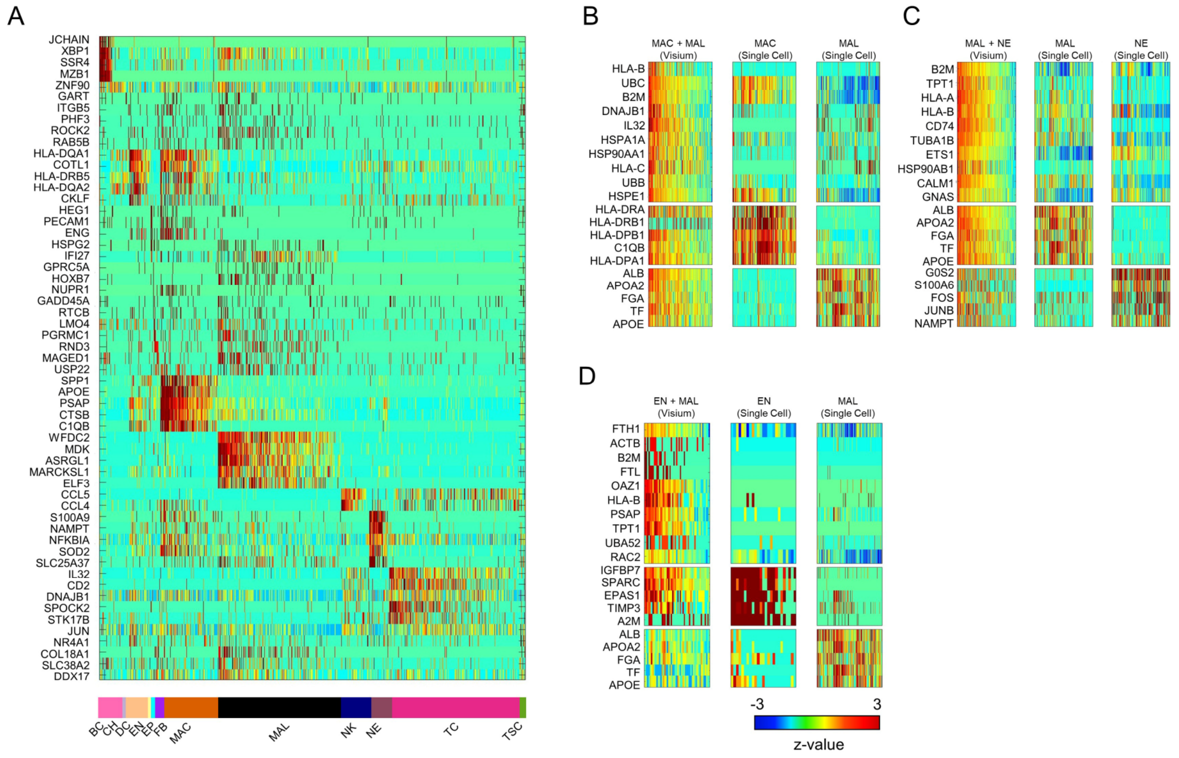

Supplementary Figure S7. NicheSVM reveals niche-specific genes in UCEC. (**A**) Heatmap visualizing cell type markers for each cell type in scRNAseq data. (**B-D**) Heatmaps depicting the top 10 niche-specific genes and their corresponding cell type markers in Visium and scRNAseq data for four major combinations; MAC+MAL (**B**), MAL+NE (**C**), and EN+MAL (**D**).

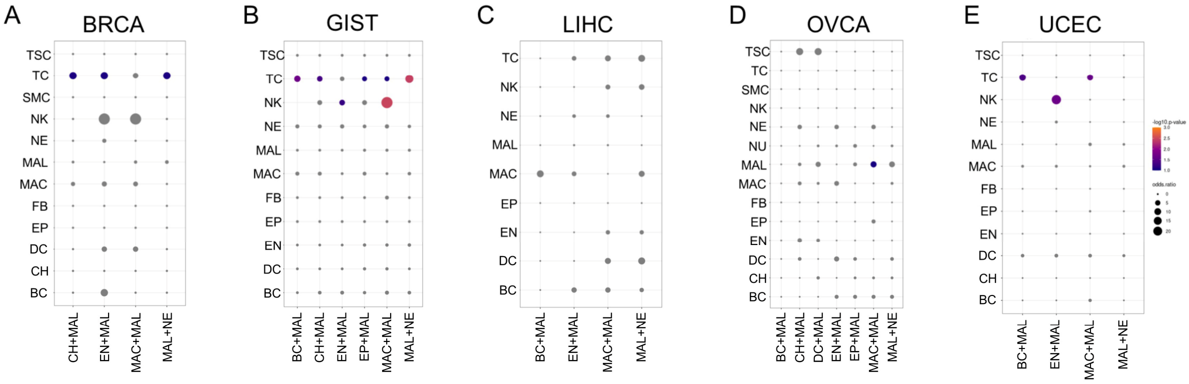

Supplementary Figure S8. The association between niche-specific genes and cell type markers across five cancer types. Dot plots showing the relevance between cell type markers and niche-specific genes of BRCA; breast cancer (**A**), GIST; gastrointestinal stromal tumor (**B**), LIHC; liver hepatocellular carcinoma (**C**), OVCA; ovarian cancer (**D**), and UCEC; uterine corpus endometrial carcinoma (**E**).

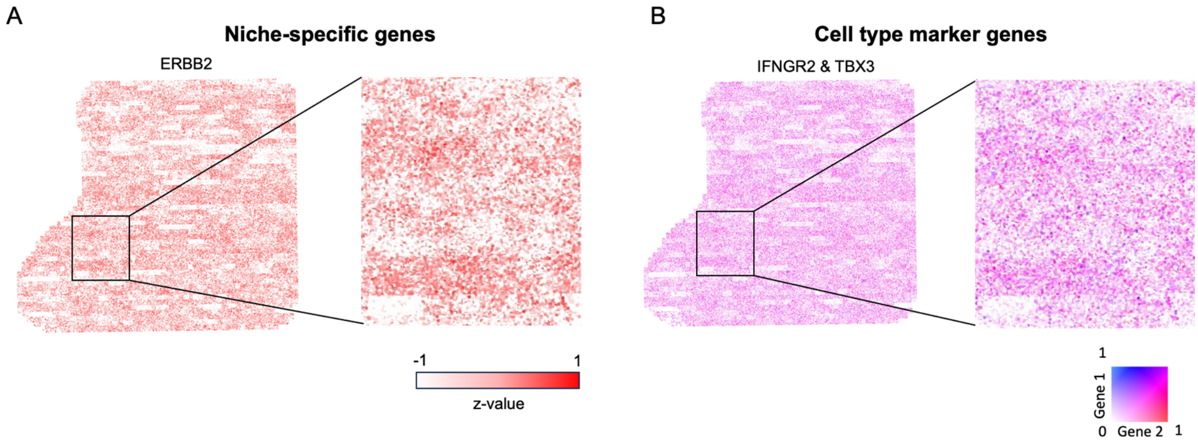

Supplementary Figure S9. Validation of a niche-specific gene using high-resolution spatial transcriptomics data MERSCOPE obtained from a breast cancer sample. ERBB2 a niche-specific gene for macrophage and malignant cell is highly expressed where both IFNGR2 a macrophage marker and TBX3 a malignant cell marker is highly expressed.

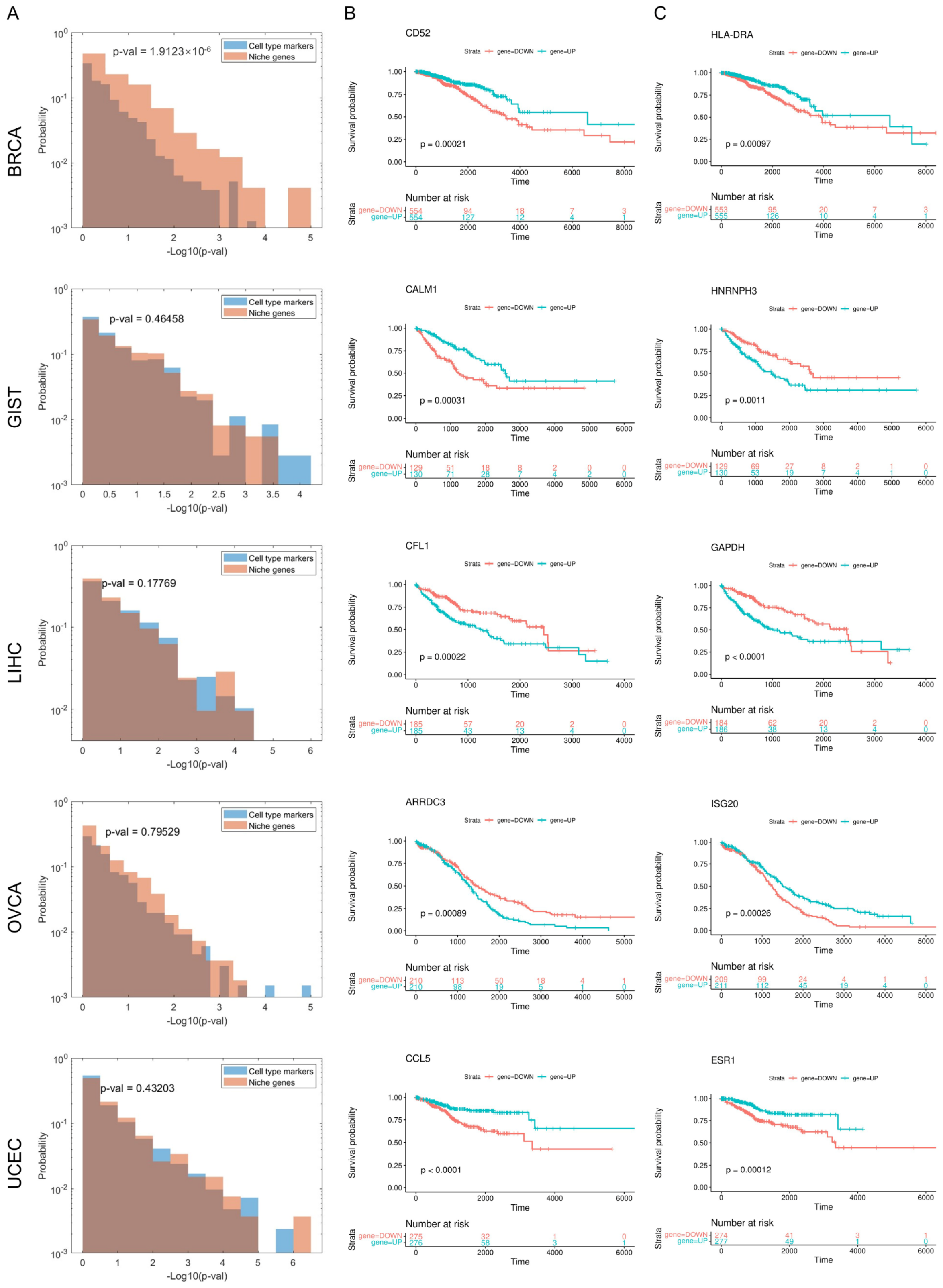

Supplementary Figure S10. Survival analysis of top 50 niche-specific genes and cell type markers across five cancer types. (**A**) Distributions of -log10(p-value) for top 50 niche-specific genes and cell

type markers. (**B**) Examples of Kaplan-Meier curves showing significant different survival rate by gene expression.

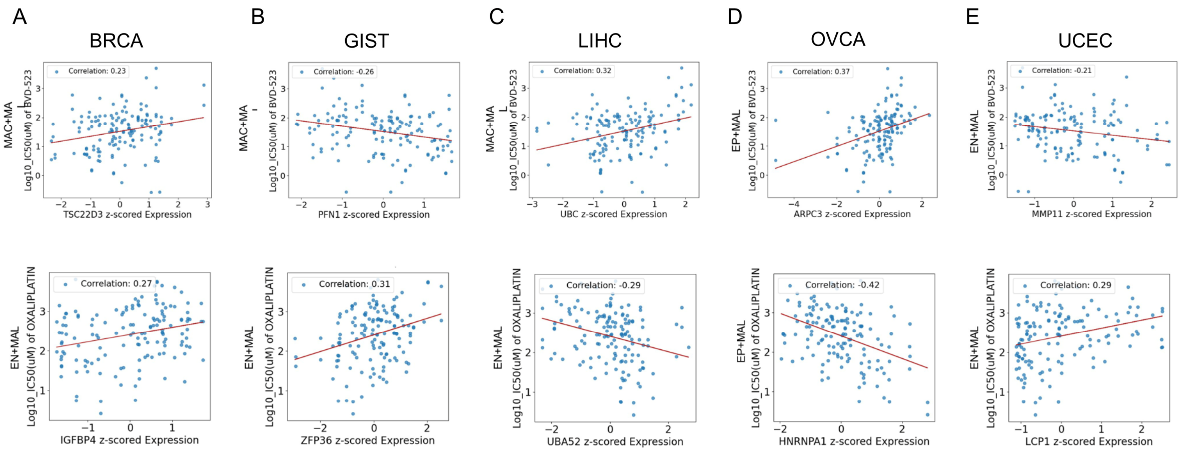

Supplementary Figure S11. Example of niche-specific genes showing high correlation with drug treatment efficiency in cancer cell line encyclopedia (CCLE) data. Scatter plots of the drug treatment efficiency of two anti-cancer drugs including BVD-523 (upper panel) and OXALIPLATIN (lower panel) and the expression level of niche-specific genes obtained from BRCA; breast cancer (**A**), GIST; gastrointestinal stromal tumor (**B**), LIHC; liver hepatocellular carcinoma (**C**), OVCA; ovarian cancer (**D**), and UCEC; uterine corpus endometrial carcinoma (**E**).

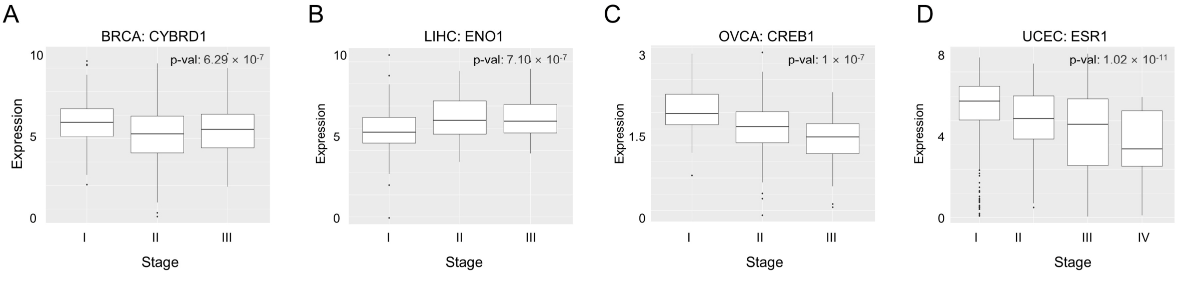

Supplementary Figure S12. Example of niche-specific genes showing association with cancer stage in the cancer genome atlas (TCGA) data. Box plots of the expression level of niche-specific genes obtained from BRCA; breast cancer (**A**), LIHC; liver hepatocellular carcinoma (**B**), OVCA; ovarian cancer (**C**), and UCEC; uterine corpus endometrial carcinoma (**D**) across cancer stages.

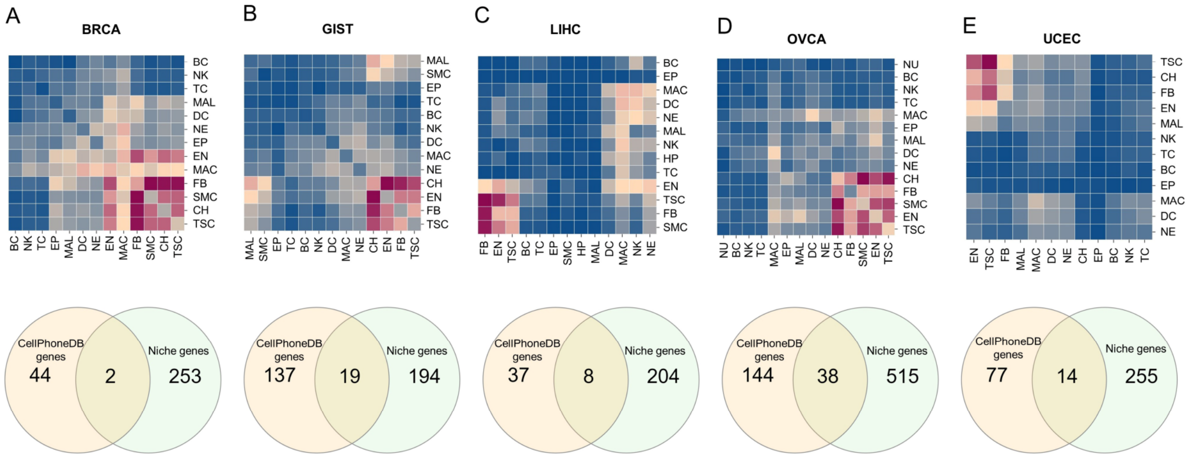

Supplementary Figure S13. Discrepancy between niche-specific genes and gene sets identified from CellPhoneDB a ligand-receptor analysis tool. Heatmaps of the intensity of cell-cell interaction between cell types (upper panel) and Venn diagrams of niche-specific genes and ligand-receptor gene set of BRCA; breast cancer (**A**), GIST; gastrointestinal stromal tumor (**B**), LIHC; liver hepatocellular carcinoma (**C**), OVCA; ovarian cancer (**D**), and UCEC; uterine corpus endometrial carcinoma (**E**).
